# Genome-Wide Analysis of MYB Transcription Factors and Screening of *MYBs* Involved in the Red Color Formation in *Rhododendron delavayi*

**DOI:** 10.3390/ijms24054641

**Published:** 2023-02-28

**Authors:** Fenfang Long, Hairong Wu, Huie Li, Weiwei Zuo, Qian Ao

**Affiliations:** College of Agriculture, Guizhou University, Guiyang 550025, China

**Keywords:** anthocyanin, chromatic aberration, RNA-seq, *Rhododendron*, transcription factor, WGCNA

## Abstract

Flower color is one of the crucial traits of ornamental plants. *Rhododendron delavayi* Franch. is a famous ornamental plant species distributed in the mountain areas of Southwest China. This plant has red inflorescence and young branchlets. However, the molecular basis of the color formation of *R. delavayi* is unclear. In this study, 184 *MYB* genes were identified based on the released genome of *R. delavayi*. These genes included 78 1R-MYB, 101 R2R3-MYB, 4 3R-MYB, and 1 4R-MYB. The *MYBs* were divided into 35 subgroups using phylogenetic analysis of the *MYBs* of *Arabidopsis thaliana*. The members of the same subgroup in *R. delavayi* had similar conserved domains and motifs, gene structures, and promoter cis-acting elements, which indicate their relatively conserved function. In addition, transcriptome based on unique molecular identifier strategy and color difference of the spotted petals, unspotted petals, spotted throat, unspotted throat, and branchlet cortex were detected. Results showed significant differences in the expression levels of *R2R3-MYB* genes. Weighted co-expression network analysis between transcriptome and chromatic aberration values of five types of red samples showed that the MYBs were the most important TFs involved in the color formation, of which seven were R2R3-MYB, and three were 1R-MYB. Two *R2R3-MYB* (DUH019226.1 and DUH019400.1) had the highest connectivity in the whole regulation network, and they were identified as hub genes for red color formation. These two *MYB* hub genes provide references for the study of transcriptional regulation of the red color formation of *R. delavayi*.

## 1. Introduction

Anthocyanins are flavonoids and the main pigments in petals and fruits. These flavonoids can give plants different colors to attract insects and help spread pollen and seeds to promote plant reproduction [1]. Anthocyanins also have anti-oxidation and anti-inflammatory activities [2]. 

Anthocyanin biosynthesis is controlled by numerous genes, such as transcription factor (TF) and structural genes. Anthocyanidin 3-O-glucosyltransferase (*UDPGT*, in short *UGT*) is a structural gene and plays a role in the anthocyanin biosynthesis of plants, such as *Capsicum annuum* [3], *Morella rubra* [4], and *Vaccinium* spp. [5]. The expression of structural genes is often regulated by TFs. MYB is one of the largest families among plant TFs. This TF can bind to cis elements in the promoters of target genes to mainly regulate plant anthocyanin biosynthesis, development, and stress resistance [6]. The N-terminus of MYB has a highly conserved DNA-binding domain (DBD). The domain contains three irregular repeats, each of which forms three A-helices. The other two helices construct a Helix-turn-Helix (HTH) structure with three evenly spaced tryptophan (Trp) residues. Hydrophobic nuclei are formed in the three-dimensional HTH structure [7]. Based on the number of repeats of domains, MYBs can be classified into 1R-MYB, R2R3-MYB, R3-MYB, and 4R-MYB [8]. Among the four types of MYB, R2R3-MYB plays the most central role in anthocyanin synthesis [9].

TFs activate/inhibit the genes that encode various catalytic enzymes to regulate anthocyanin biosynthesis [10]. MYB alone, or by forming a MBW complex with basic helix-loop-helix (bHLH) and WD40, regulates anthocyanin biosynthesis by binding to specific regions of the target gene promoter [11]. The function of R2R3-MYB in regulating anthocyanin biosynthesis has been reported in numerous species. In apple fruit, *MdMYB306* interacts with *MdMYB17* and *MdbHLH33* through its N-terminal to inhibit anthocyanin synthesis and affect fruit color [12]. In *Pistacia chinensis*, *PcMYB113* makes the leaves red by promoting anthocyanin biosynthesis during autumn leaf coloration [13]. MYB also regulates the anthocyanin biosynthesis in peony [14], pear [15], pomegranate [16], and kiwi fruit [17].

High-throughput RNA sequencing (RNA-Seq) can generate information-rich sequence and expression data sets to characterize the abundance of related genes in plants [18,19]. RNA-Seq using a unique molecular identifier (UMI) strategy can eliminate quantitative interference by polymerase chain reaction (PCR) amplification preferences, leading to more accurate expression results [20,21]. Hub genes associated with a certain trait can be identified using the weighted gene co-expression network analysis (WGCNA) of RNA-Seq genes based on the UMI strategy associated with trait phenotypic data [22,23,24].

*Rhododendron delavayi* is a small evergreen tree belonging to the subgenus *Hymenanthes* (*Rhododendron*, Ericaceae). This species is widely distributed throughout Southwest China with large inflorescences, and it is an important dominant species in the nature reserve. As one of the world’s famous ornamental plants, the inflorescence of *R. delavayi* consists of more than 10 small flowers. The flowers and branchlets of this species are all red and contain high concentrations of anthocyanins [25]. Previous studies have shown that overexpression of *RdDFR1*, *Rd3GT1*, and *Rd3GT6* changed the transgenic tobacco flower color from light pink to dark pink [26,27]. However, no TF genes have been reported except for the structural genes related to the anthocyanin biosynthesis of *R. delavayi*.

Therefore, in view of the vital regulatory role of MYBs in the anthocyanin biosynthesis of other plants, in this study, the MYB TF family was analyzed based on the released *R. delavayi* genome [28]. In addition, the expression profiles of the members of this family were detected by RNA-Seq using the UMI strategy on five types of colorful samples, namely, spotted petals, unspotted petals, spotted throat, unspotted throat, and branchlet cortex. With the WGCNA of the transcriptome and color difference values, the hub genes related to the red color formation of *R. delavayi* were identified. The data will provide an important reference for the study of the transcriptional regulation mechanism of the color formation of *R. delavayi*.

## 2. Results

### 2.1. Genome-Wide Identification of the Members of R. delavayi MYB Gene Family

**Figure 1:**
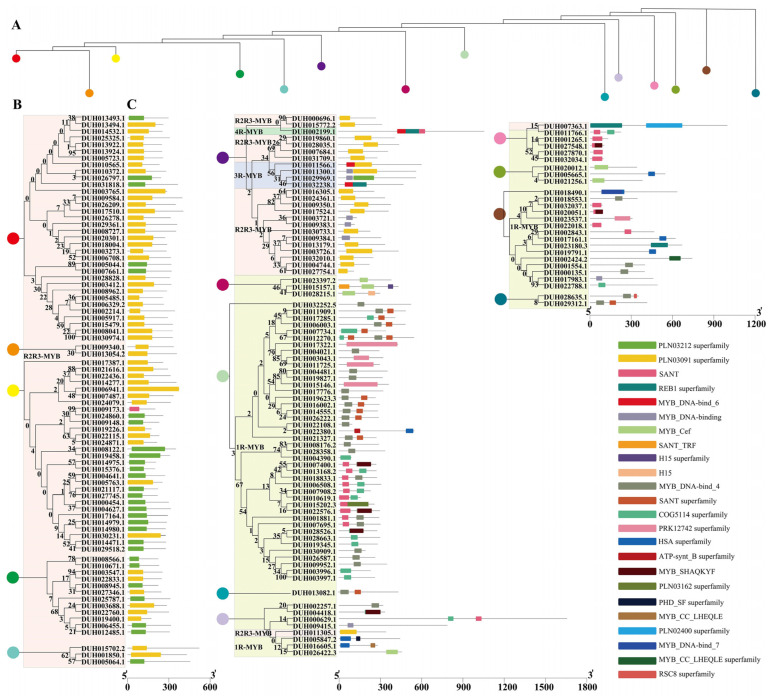
Conserved MYB domain in MYBs of *Rhododendron delavayi.* (**A**) Phylogenetic tree trunk of 184 *R. delavayi* MYB genes. It was constructed by maximum likelihood (ML) method based on multiple sequence alignment of 184 amino acid sequences of *R. delavayi* MYB gene. Different colored circles indicate each node of the phylogenetic tree. (**B**) Branches on each node of the trunk of the phylogenetic tree. The MYB domain was divided into 1R-MYB, R2R3-MYB, 3R-MYB, and 4R-MYB and marked with a colored background. (**C**) Conserved domain composition of these genes. The colored squares indicate the different structural domains.

The whole released genome of *R. delavayi* [28] was analyzed to identify the MYB gene family. A total of 202 *MYB* genes were selected by BLAST against the genome by using the protein sequences of 168 *MYB* genes in *Arabidopsis thaliana*. In addition, 292 *MYB* genes were hit by using the Hidden Markov Model (HMM) to query the MYB DBD (PF00249). Combining the blast results of the two different methods, a total of 184 MYB members were finally identified, including 78 1R-MYB, 101 R2R3-MYB (2R-MYB), 4 R1R2R3-MYB (3R-MYB), and one 4R-MYB protein (Figure 1) as analyzed against Pfam, InterPro, and Prosite databases. The isoelectric point of the identified MYBs ranged from 4.45 (DUH002843.1) to 10.42 (DUH004390.1), and the predicted molecular weights ranged from 7412.46 kDa (DUH004390.1) to 179,531.3 kDa (DUH000629.1) (Table S1). In addition, subcellular localization prediction of MYBs showed that 175 of the 184 MYB proteins were located in the nucleus, five in the cytoplasm, and four in mitochondria (Table S1).

### 2.2. Analysis of the MYB Conserved Motif and Gene Structure of R. delavayi

**Figure 2:**
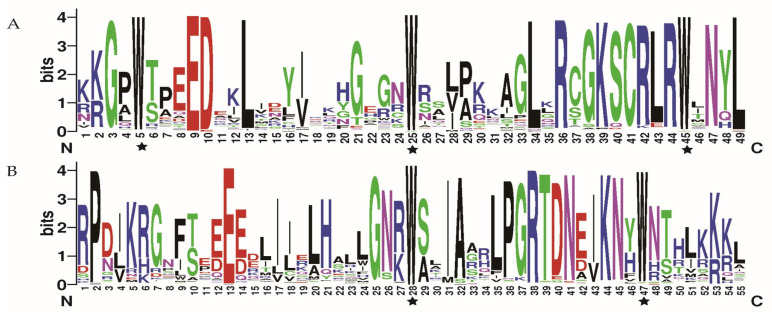
R2 and R3 MYB repeats of R2R3-MYBs are highly conserved in *Rhododendron delavayi*. R2 (**A**) and R3 (**B**) MYB repeat sequences were based on multiple alignment analysis of the R2R3-MYB structural domain of all *R. delavayi*. Bits indicate the relative frequency of amino acids. Asterisks indicate conserved Trp residues in the MYB domain. The amino acids are arranged from the N terminal to the C terminal.

**Figure 3:**
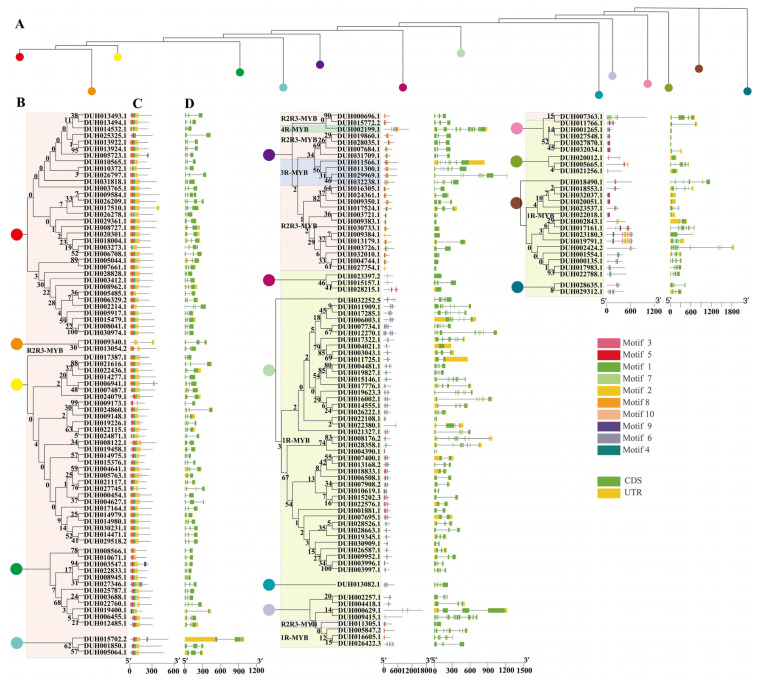
Motif composition and gene structure of *Rhododendron delavayi* MYB genes. (**A**) Phylogenetic tree trunk of 184 *R. delavayi* MYB genes. Different colored circles indicate each node of the phylogenetic tree. (**B**) The MYB structural domains on each node branch were divided and marked with colored backgrounds. (**C**) Conservation motifs of the MYB proteins. Each motif represents a number in the colored box, and a black line represents the non-conserved sequence. (**D**) Gene structure of MYB. Green, yellow, and black colors represent the UTR, exon, and intron, respectively. The scale bar at the bottom indicates the length of motifs and genes, respectively.

To study the sequence characteristics of the conserved MYB DBD of *R. delavayi*, we performed multiple sequence alignments on their amino acid sequences. The MYB repeats on R2 and R3 of *R. delavayi* contained characteristic amino acids (Figure 2). Three highly conserved Trp residues were found at positions 5, 25, and 45 in the R2 repeats of R2R3-MYB, whereas two Trp residues were found at positions 28 and 47 in R3 (Figure 2). The phylogenetic tree was constructed by the ML method using the 184 MYB protein sequences of *R. delavayi*. The MYB family was clustered into different branches (Figure 3). Ten conserved motifs of MYB proteins were identified by the MEME program. Among all MYBs, R2R3-MYB, 3R-MYB, and 4R-MYB genes had high similarity in the amino acid sequences (Figure 3A). The 1R-MYB protein contained motifs 3 and 4. Other types of MYB proteins basically contained motifs 1, 2, 3, and 5 (Figure 3A). The MYB proteins of *R. delavayi* in the same group had similar motif types and quantities, which indicates that these proteins may have similar functions. 

To further measure the structural characteristics of *R. delavayi MYB* genes, we further analyzed their introns/exons. The most homologous genes in a group shared the same gene structure layout and the number of exons and introns (Figure 3B). All 3R-MYB contained seven exons, whereas 4R-MYB contained 10 exons. R2R3-MYB contained one–four exons, 68% of which contained conserved gene structure with three exons and two introns. In addition, 1R-MYB contained 1–13 exons, 51% of which had more than four exons. Some introns of the genes were large and accounted for a big part of the genes. Compared with CDS sequences, most MYB genes are with or without two untranslated regions (UTRs). The UTR sequences are less similar, and their diversity is expressed in terms of gene length, which indicates that UTR sequences are less conserved than the coding regions.

### 2.3. Phylogenetic Analysis of the MYB Family of R. delavayi

**Figure 4:**
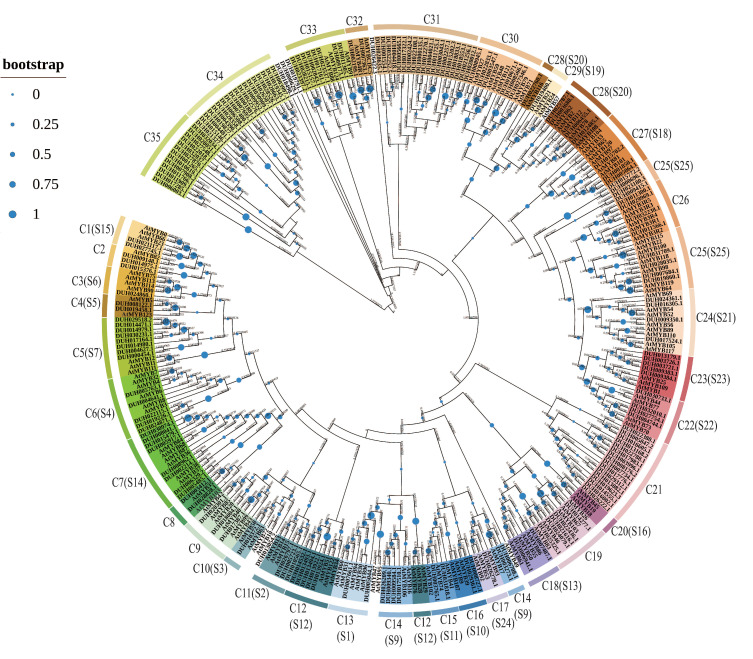
Phylogenetic tree of MYB proteins between Arabidopsis thaliana and *Rhododendron delavayi* (S). ML phylogeny was determined by MEGA7.0. Phylogenetic tree was divided into 35 main groups. Different colored clusters are labeled outside the tree, whereas their subgroups are marked with the corresponding color backgrounds. The blue circles on the branches are bootstrap and the numbers are branch lengths.

To study the evolutionary relationship of MYB genes in *R. delavayi*, we downloaded 132 protein sequences of *A. thaliana* MYBs from TAIR, together with 184 protein sequences of *R. delavayi* MYBs, and constructed a ML phylogenetic tree. The MYB members of *R. delavayi* were divided into 35 subgroups and named C1 to C35 based on sequence similarity and topological structure, according to the classification of the same family in *A. thaliana*. The clades (S1–S25 subgroups) were labeled based on the evolutionary relationship in *A. thaliana* in the tree (Figure 4). The phylogenetic tree showed that most of the *R. delavayi* MYBs were clustered with R2R3-MYB of *A. thaliana*. Subgroup C31 contained a maximum of 19 members, whereas subgroup C20 had none. Four subgroups, namely, C21, C31, C34, and C35, were not grouped with the MYB of *A. thaliana.* The results suggest that they may have undergone gene gain or loss events during evolution.

### 2.4. Analysis of R. delavayi MYB Genes Promoters Cis-Acting Elements

**Figure 5:**
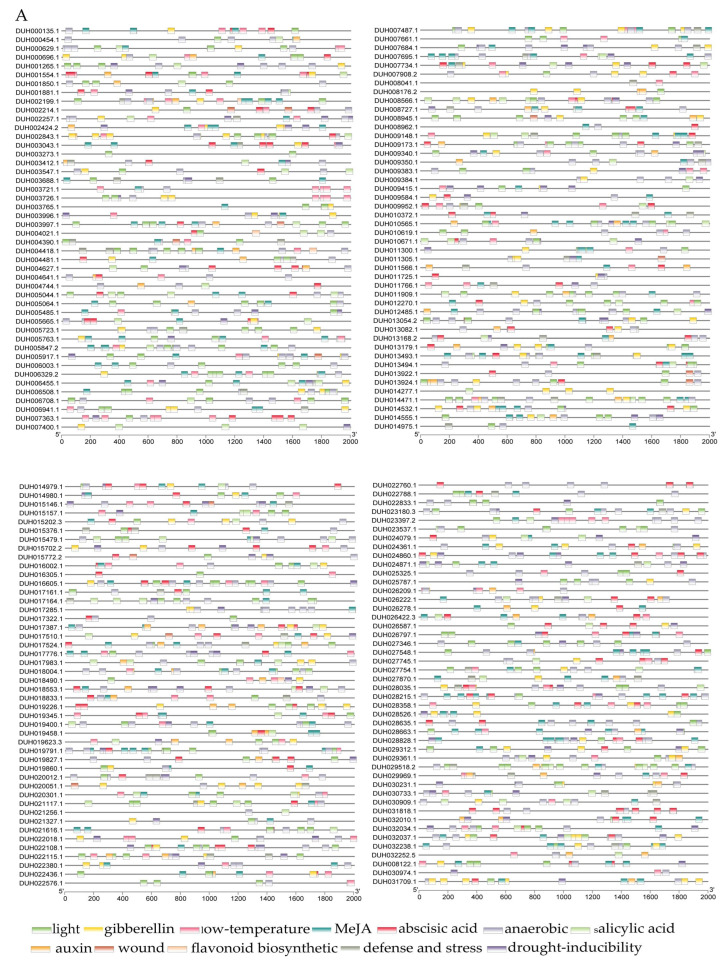

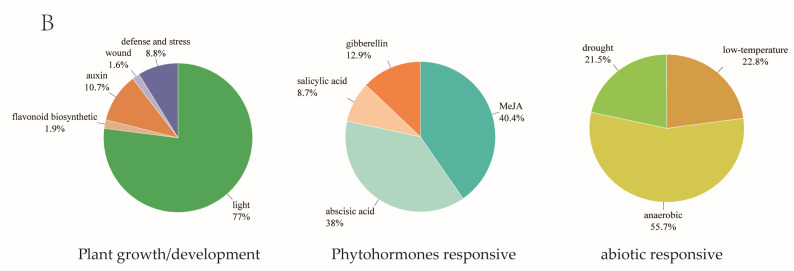
Cis-acting elements in the MYB promoter region of *Rhododendron delavayi*. (**A**) MYB gene promoter region regulatory elements. Boxes of different colors represent various cis elements and are distributed in different positions among 184 MYB genes. Axes represent gene length. (**B**) Statistics of cis elements in MYB promoter involved in plant growth and development, phytohormone response, and abiotic stress response.

Cis-acting elements in the promoter region of *R. delavayi MYB* genes can be divided into three categories: plant hormone, biological/abiotic stress, and plant growth and development response elements (Figure 5 and Table S2). Plant hormone response elements, such as Me-JA response elements (e.g., CGTCA and TGACG motifs), abscisic acid response elements (ABRE), gibberellin response elements (e.g., P-box), and salicylic acid response elements (e.g., TCA element), were widely distributed in MYB promoters. The Me-JA elements accounted for the highest proportion (40%). Cis elements involved in abiotic stress responses included anaerobic inducible elements (ARE), low-temperature response elements (LTR), and drought-inducible elements (e.g., MBS). Among the growth and development response elements, the light (e.g., G-Box) and auxin response elements (e.g., TGA element) accounted for 77% and 11%, respectively. The defense and stress response elements (e.g., TC-rich repeats) accounted for 9%, but the trauma response elements (e.g., WUN motif) accounted for 1%. Flavonoid biosynthesis response elements (e.g., MBSI) accounted for 2%. All cis elements appeared most frequently in the promoters of 2R-MYB, and different subgroups of MYB promoters had distinct cis element types. One TCA element and one WUN motif were found in 1R-MYB and 2R-MYB promoters, respectively, which indicates the functional diversity of MYB in *R. delavayi*. Moreover, a total of 101,112 TF-binding sites and 223 potentially interacting TFs described previously in *A. thaliana* were revealed using the PlantPan 3.0 (Table S3). MYB binding sites were identified at all MYB promoters examined. There were also several binding sites of TFs other than the MYB family on the examined promoters, such as the binding sites of bZIP, bHLH, and WRKY.

### 2.5. Transcriptomic Analysis

**Figure 6:**
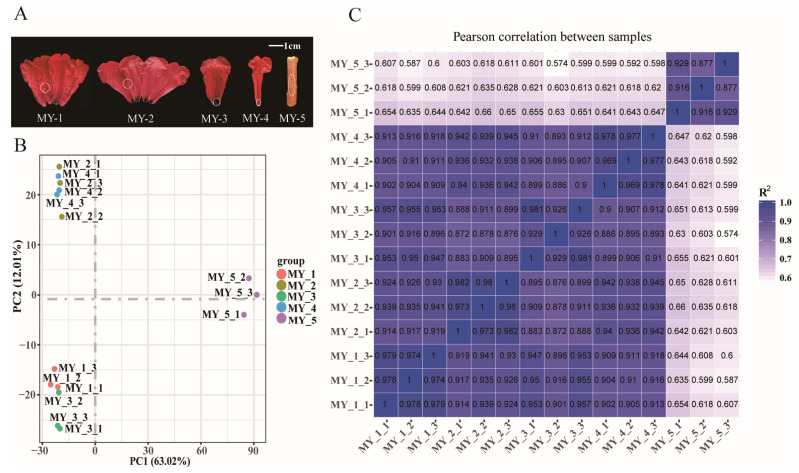
Basic transcriptome data of five types of red samples of *R. delavayi* spotted petals, unspotted petals, spotted throat, unspotted throat, and branchlet cortex. (**A**) Phenotypes of five types of samples of *R. delavayi*: MY-1, spotted petals; MY-2, unspotted petals; MY-3, spotted throat; MY-4, unspotted throat; MY-5, branchlet cortex. The enclosed part represents the sampling site. (**B**) PCA analysis of the five types of samples; (**C**) Pearson correlation coefficient of the five types of samples.

To determine the MYB genes that play a role in the color formation of *R. delavayi*, we performed RNA-seq analysis based on the UMI strategy using spotted petals, unspotted petals, spotted throat, unspotted throat of flower, and branchlet cortex (Figure 6A). The produced raw data of the transcriptome have been uploaded to the Sequence Reading Archive database (http://www.ncbi.nlm.nih.gov/sra/, accessed on 3 December 2022) with entry number PRJNA907866. After the removal of low-quality reads and adapters, an average of 23,879,787 clean reads were obtained per sample. The percentage of bases with a phred value greater than 20 in the total bases (QC20), the percentage of bases with a phred value greater than 30 in the total bases (QC30), and GC percentage were greater than 97%, 92%, and 47%, respectively (Table S4). In these clean reads, 41,484,380 (90.77%) were mapped to the *R. delavayi* reference genome (Table S5). Fragments per kilobase of exon models per million mapped fragments (FPKM) were used to determine the expression level of unigenes. Based on the FPKM values, principal component analyses (PCA) and Pearson correlation coefficients showed high correlation coefficients between three biological replicates (Figure 6B,C, respectively).

### 2.6. Differentially Expressed Genes (DEGs), Gene Ontology (GO), and Kyoto Encyclopedia of Genes and Genomes (KEGG) Analysis

**Figure 7:**
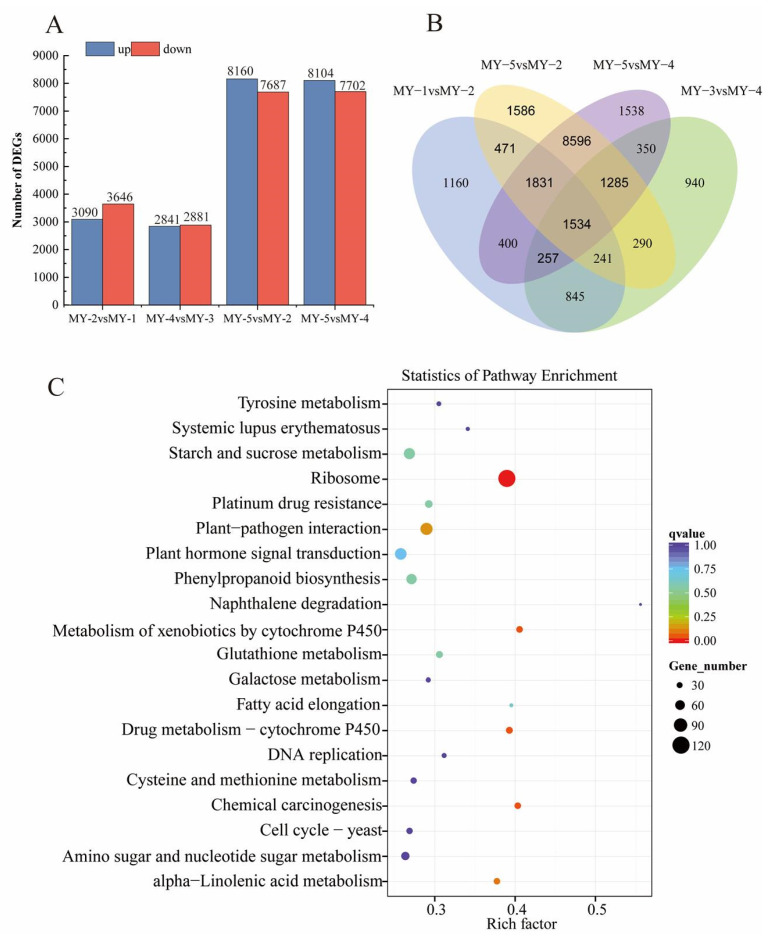
RNA-Seq analysis of *R. delavayi*. (**A**) Histograms of DEGs obtained by comparison of four pairs of samples, namely, MY-1 vs MY-2, MY-3 vs MY-4, MY-5 vs MY-2, and MY-5 vs MY-4, respectively. (**B**) Venn diagram of DEGs in the four combinations. (**C**). KEGG enrichment analysis of MY-1 vs MY-2 DEGs.

The DEGs and shared genes between samples were analyzed. Comparative analysis showed 6736 DEGs between MY-1 and MY-2 (3090 up-regulated genes and 3646 down-regulated genes) (Figure 7A); 5722 DEGs between MY-3 and MY-4 (2841 upregulated and 2881 downregulated) (Figure 7A); 15,847 DEGs between MY-5 and MY-2 (8160 upregulated and 7687 downregulated) (Figure 7A); 15,806 DEGs between MY-5 and MY-4 (8104 upregulated and 7702 downregulated) (Figure 7A). In addition, the Venn diagram showed the following: 1160 genes shared expressions in MY-1 and MY-2; 940 genes shared expressions in MY-3 and MY-4; 1586 genes shared expressions in MY-5 and MY-2; 1538 genes shared expressions in MY-5 and MY-4 (Figure 7B). GO and KEGG enrichment analyses of different sample combinations revealed that these DEGs are related to various metabolic and biosynthetic pathways (Figure S1 and Figure Figure 7C), and the DEGs of all four combinations were enriched in the pathways encoding phenylpropanoid biosynthesis (ko00940), flavonoid biosynthesis (ko00941), and anthocyanin biosynthesis (ko00942). Thus, DEGs may be important for anthocyanin biosynthesis in *R. delavayi*.

### 2.7. Expression Analyses of R2R3-MYB Genes in R. delavayi

**Figure 8:**
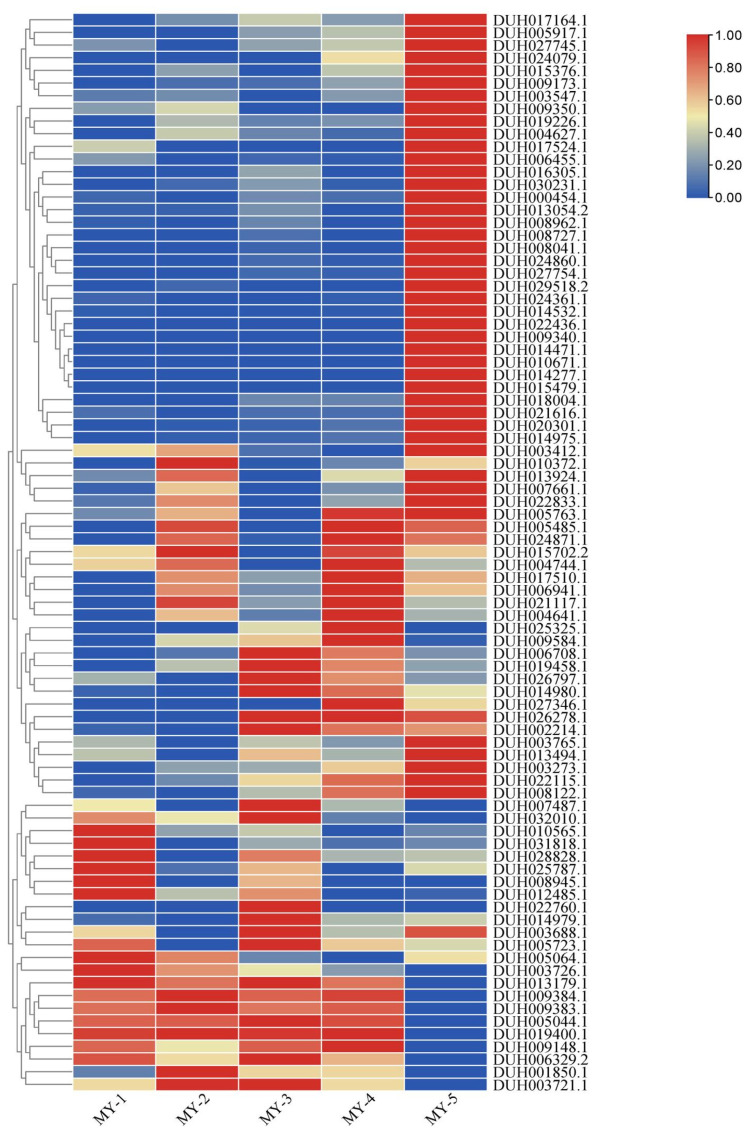
Expression heat maps of 86 R2R3-MYB genes in different samples of *R. delavayi*: MY-1, spotted petals; MY-2, unspotted petals; MY-3, spotted throat; MY-4, unspotted throat; MY-5, branchlet cortex. The genes on the right indicate the differentially expressed R2R3-MYB in the five types of red samples. Blue rectangles indicate low expression levels, whereas red rectangles indicate high expression levels. Generated hierarchical clustering tree maps and heat maps based on log_2_ values of FPKM.

The MYB TFs play an important role in anthocyanin biosynthesis, especially R2R3-MYB. To evaluate the expression pattern of R2R3-MYB genes in anthocyanin biosynthesis in different tissues of *R. delavayi*, we evaluated the expression levels of R2R3-MYB genes based on the transcriptome data of spotted petals, unspotted petals, spotted throat, unspotted throat, and branchlet cortex. A total of 91 R2R3-MYB genes were identified, and 85 of them showed differential expression in five types of red samples (Figure 8). Among these genes, 10 were significantly expressed in four tissues, including spotted petals, non-spotted petals, spotted larynx, and non-spotted larynx, whereas 34 were significantly expressed only in the branchlet cortex. The expression of certain genes was limited to certain tissues, such as MYB genes DUH010372.1 and DUH007487.1 which were only significantly expressed in unspotted petals and spotted throat, respectively. Heat maps of R2R3-MYB expression in *R. delavayi* showed that most MYB genes were expressed differently in the five types of samples, and several genes were expressed only in the branchlet cortex.

### 2.8. Analysis of Co-Expression Network

**Figure 9:**
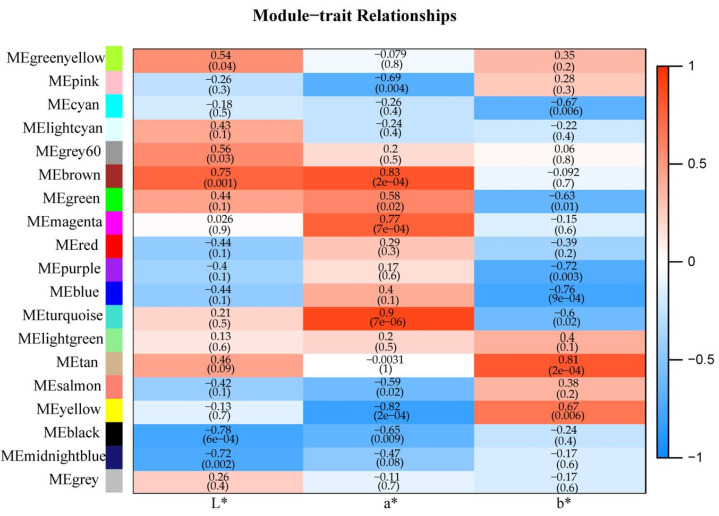
Correlation analysis of WGCNA module with chromatic aberration. Each column corresponds to L*, a*, and b* values, and each row represents a module. Chromatic aberration values and module correlation coefficients are shown within each module. *p*-values are shown in parentheses.

**Figure 10:**
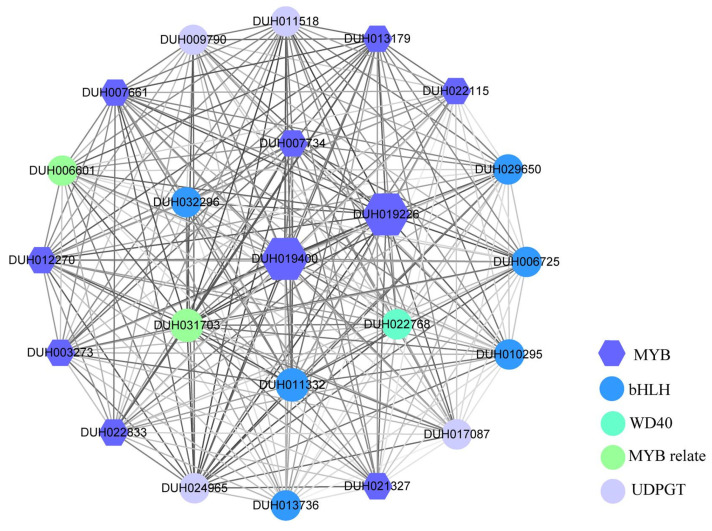
Visualization of hub genes in the MEturquoise module related to the a* value. Different color lumps represent distinct families, with a larger shape for a greater degree and a darker line for a greater weight.

Tissue colors were determined using the CIE 1976 (L*a*b*) system [29], that is, the larger the value of L*, the brighter the color, and the smaller the value, the darker the color; the larger the value of a*, the more reddish, and the smaller the value, the more greenish the tissue; the larger the value of b*, the more yellowish, and the smaller the value, the more bluish the tissue [30,31]. To screen hub genes regulating the red-color formation of *R. delavayi*, the correlation of color indicators of L*, a*, and b* values (Table 1) and the gene expression levels based on the RNA-Seq data of the five types of red samples were analyzed using WGCNA. A total of 19 co-expression modules were detected in the screened unigenes (Figure 9). Modules ranged in size from 45 unigenes (gray module) to 5225 unigenes (turquoise module). For the a* value, the a* indicator represents the transition from red to green [30,31], which was positively correlated with the MEturquoise module, with the highest correlation and strongest significance (r = 0.9, *p* = 7 × 10^−6^). A total of 23 highly connective genes (degree ≥ 4962), including 10 MYBs, six bHLHs, one WD40 gene, two MYB-relate genes, and four structural genes (UDPGT) were screened in the module (Figure 10). Among the 10 MYBs, seven were R2R3-MYB genes (DUH019226.1, DUH019400.1, DUH013179.1, DUH007661.1, DUH003273.1, DUH022833.1, and DUH022115.1), and three were 1R-MYB genes (DUH007734.1, DUH012270.1, and DUH021327.1). These results indicate that the MYBs, bHLHs, WD40, and UDPGT identified are probably involved in the red color formation of *R. delavayi*. In addition, the co-expression network showed that two R2R3-MYBs (DUH019226.1 and DUH019400.1) had the highest connectivity in the whole regulation network and were identified as hub genes for red color formation. The identified bHLH, WD40, and UDPGT genes may also be involved in red color formation in *R. delavayi*.

### 2.9. Quantitative Reverse Transcription qRT-PCR Analysis of Gene Expression

**Figure 11:**
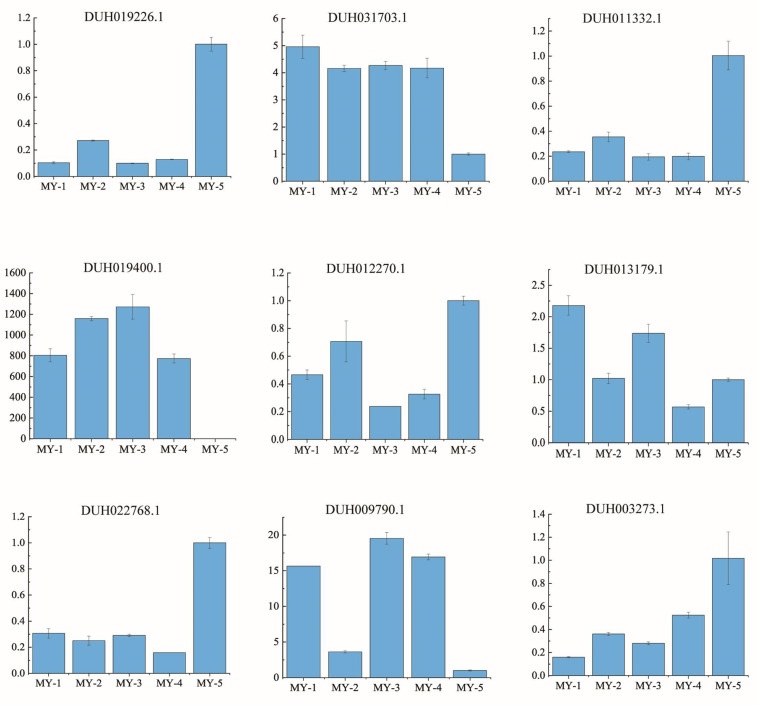
qRT-PCR verification of the transcriptome result. Expression levels of nine genes in different tissues: MY-1, spotted petals; MY-2, unspotted petals; MY-3, spotted throat; MY-4, unspotted throat; MY-5, branchlet cortex. The 18s gene was applied as a reference gene. Relative gene expression was calculated with MY-5 as control. Each column represents the mean ± standard error of the expression of three independent experiments with three replications.

Based on the co-expression network analysis, nine genes were randomly selected to further verify the accuracy of transcriptome results. Fold changes in the different tissues relative to the branchlet cortex were analyzed using qRT-PCR. The results showed that all genes detected by qRT-PCR had expression patterns similar to those of transcriptome data, and the relative expression was significantly different in petal and vegetative tissues (Figure 11). UDPGT (DUH009790.1) and MYB (DUH013179.1) were differentially expressed in all five types of red samples. Meanwhile, MYB (DUH019226.1, DUH012270.1, and DUH003273.1), bHLH (DUH011332.1), and WD40 (DUH022768.1) had high relative expression levels in the branchlet cortex. However, MYB-related genes (DUH031703.1) and MYB (DUH019400.1) had high expression in flower tissues, including spotted petals, unspotted petals, spotted throat, and unspotted throat. These qRT-PCR results further confirmed the gene repression reliability of the transcriptome.

## 3. Discussion

MYB TFs are one of the largest gene families in plants and play a crucial role in anthocyanin biosynthesis. This family has been systematically studied in *A. thaliana*, rice, tomato, petunia, and kiwi fruit [8,32,33,34,35,36]. A total of 184 members of the MYB family were identified in *R. delavayi.* This family has more members in *R. delavayi* than in rice (155) and *A. thaliana* (168), whereas the number was lower than those in peach (256), apple (229), and other plant species [37,38]. Overall, the number is greater than 100 for different species, which indicates that this family is greatly amplified in higher plants [39]. In *R. delavayi*, the R2R3-MYB subfamily is the most abundant in the MYB family, consistent with previous studies that reported 110 members of R2R3-MYB in rice [40] and 126 members in *A. thaliana* [9]. In this study, the majority of R2R3-MYB genes (68%) in *R. delavayi* contained a conserved gene structure with three exons and two introns, which are also found in other plant species [41]. Most homologous genes in the same group of the family have a common exon/intron structure and exon number, which indicates a common evolutionary history. Besides, some introns of the genes were large and accounted for a big part of the genes, the length of introns sometimes are also related to some process of adaptation in evolution [42].

Phylogenetic analysis revealed clustered homologous genes in the same clades and subclades, which often exhibit similar functions [43]. The phylogenetic trees constructed from 132 *A. thaliana* MYBs and 184 *R. delavayi* MYBs further illustrated the evolutionary relationship of *R. delavayi* MYBs. Based on the classification of MYB in *A. thaliana* [9], the MYB members of *R. delavayi* were divided into 35 subgroups (C1–C35) (Figure 4), among which four subgroups (C21, C31, C34, and C35) of *R. delavayi* did not cluster with the MYBs of *A. thaliana*. This result indicated that the six subgroups may have acquired or lost genes during evolution compared with *A. thaliana*. In addition, most of the clades had different numbers of *A. thaliana* MYBs and *R. delavayi* MYBs, and genes from the same clades may share a common evolutionary process. Based on the reported classification and function of MYBs in *A. thaliana*, phylogenetic trees would be helpful to predict the function of *R. delavayi* MYB. R2R3-MYBs of *A. thaliana* in the subclades of S3, S4, S5, S6, and S7 regulated phenylpropanoid biosynthesis [8]. This result indicated that *R. delavayi* MYBs clustered in the same subclades may also be involved in regulating phenylpropanoid biosynthesis.

Transcriptome analysis had been widely used to study gene regulation in horticultural traits [43,44,45]. In this study, KEGG analysis showed that the petals and flower throat had enriched genes in the pathway of phenylpropanoid and flavonoid biosynthesis. In addition, R2R3-MYB TFs in the reported plant species, such as *A. thaliana* [8], strawberry [46], and kiwi fruit [47], are involved in the activation of structural genes related to phenylpropanoid biosynthesis pathway and regulation of biosynthesis and accumulation of anthocyanins. Similarly, in this study, 86 R2R3-MYB TFs of *R. delavayi* were differentially expressed in the differently colored tissues. Ten R2R3-MYB genes were significantly expressed in the tissues from spotted petals, unspotted petals, spotted throat, and unspotted throat. Several R2R3-MYB genes were expressed only in specific tissues, which suggests that these R2R3-MYB genes play a key role in various aspects of color formation or the development of *R. delavayi* [34]. In addition, an analysis of cis-acting elements in *R. delavayi* showed that almost all MYB genes contained cis-acting elements, which respond to hormone signal transduction, abiotic stress, and plant growth and development. This result is consistent with previous studies on *Petunia* [34] and *Actinidia*, in which MYB promoters also contained these cis-acting elements [47].

TFs play an important role in color formation by regulating the temporal and spatial expression of structural genes [48]. MYB can form MBW complexes with bHLH and WD40 to activate or inhibit the transcription of target genes to regulate anthocyanin synthesis [48,49]. *MdMYB10* affects the anthocyanin biosynthesis of *Malus spectabilis* by regulating the expression of *ANS* [50]. *PsMYB10.2* promotes anthocyanin accumulation in *Prunus salicina* fruits by activating *PsUFGT* and *PsGST* [51]. *GbBM* of *Gossypium barbadense* encoding *MYB113* directly targets the promoter of four flavonoid biosynthesis genes and positively regulates the development of petal spots [52]. The interaction between *AcMYB10* and *AcbHLH5* in *A. chinensis* promotes the expression of *AaF3H* and *AaF3G*, respectively, and this process is conducive to anthocyanin accumulation in the flesh [53]. These findings suggest that MYBs are also widely involved in the regulation of anthocyanin biosynthesis in non-model plant species. In addition, WGCNA showed that *DcMYB113* of *Daucus carota* activates the expressions of *DcbHLH3* and anthocyanin biosynthesis-related structural genes [54]. Eleven genes (e.g., *PaMYB10* and seven structural genes) were identified in the *Prunus armeniaca* co-expression network. PaMYB10, MdMYB10, and PsMYB10 belong to the anthocyanin-associated R2R3-MYB branch, showing high homology [55]. Similar to previous reports, *GbBM* and *DcMYB113* belong to the S6 clade of the R2R3 MYB. In this study, WGCNA showed that 10 *MYBs*, six *bHLHs*, one *WD40*, two MYB-related genes, and four *UDPGTs* genes constructed the co-expression network behind the coloration in *R. delavayi*. Among these genes, two R2R3-MYB TF genes (DUH019400.1 and DUH019226.1) were identified as hub genes associated with anthocyanin biosynthesis in *R. delavayi*. qRT-PCR analysis revealed that the MYB TF DUH019226.1 was significantly expressed in the branchlet cortex, and DUH019400.1 was significantly expressed in the tissues of the remaining four petals. DUH019226.1 in the phylogenetic tree clustered into the S4 subgroup related to anthocyanin synthesis [5]. DUH019400.1 was clustered into the S19 subgroup, which controls another development or function [48]. The results indicate the possible gene acquisition or loss events during the evolution of *R. delavayi* MYBs.

## 4. Materials and Methods

### 4.1. Plant Materials and Color Determination

Plant samples of *R. delavayi* were collected from Baili Rhododendron Reserve, Guizhou Province, China. The samples were obtained from five types of red samples, namely, spotted petals, unspotted petals, spotted throat, unspotted throat, and branchlet cortex. The samples were randomly collected with three biological replicates. After sampling, the samples were immediately frozen in liquid nitrogen and stored at −80 °C. Color deference was represented using the CIE 1976 (L*a*b*) system, the color values of all samples were determined by a portable color spectrometer (CHN Spe, Hangzhou, China) before sampling, and the values of L*, a*, and b* were determined with three replicates.

### 4.2. Identification and Sequence Analysis of the MYB Gene Family

Whole released genome sequence of *R. delavayi* was downloaded from the Rhododendron Plant Genome Database (RPGD) (http://bioinfor.kib.ac.cn/RPGD/index.html, accessed on 1 October 2022) [28]. The protein sequences of 168 MYBs of *A. thaliana* were downloaded from the Plant Transcription Factor Database (http://planttfdb.gao-lab.org/, accessed on 3 October 2022) [8]. All MYBs of *R. delavayi* were identified using blastp (E < 1 × 10^−5^) with MYBs of *A. thaliana* queries, which is available from the NCBI (http://www.ncbi.nlm.nih.gov/, accessed on 3 October 2022). The DBD (PF00249) of MYB was downloaded from the Pfam database (http://pfam.sanger.ac.uk/, accessed on 4 October 2022) and used as a query. HMMER (http:/hmmer.janelia.org/, accessed on 4 October 2022) version 3.1b2 software was used to search and identify the MYB candidate sequence in the database of *R. delavayi*, and the cut-off of E value was set to <1.0 × 10^−5^ [56]. Then, the intersection of results from the two different methods elucidated common elements. To verify the reliability of the results, candidate MYB proteins were further analyzed using Pfam, InterPro (http://www.ebi.ac.uk/interpro/, accessed on 5 October 2022), and PROSITE [57] (https://prosite.expasy.org/, accessed on 5 October 2022). Sequences lacking the central or entire MYB DBDs were filtered out. The selected MYB proteins of *R. delavayi* molecules (molecular weight and isoelectric point) were calculated through Expasy (https://web.expasy.org/compute_pi/, accessed on 7 October 2022). Subcellular localization of MYBs was predicted using Cello (http://cello.life.nctu.edu.tw, accessed on 7 October 2022) [58].

### 4.3. Analysis of the MYB Conserved Motif and Gene Structure

The gene structure of *MYBs* was analyzed using TBtools software v1.0987663 (https://github.com/CJ-Chen/TBtools/releases, accessed on 12 October 2022). MYB exon/intron was visualized using the TBtools [59]. The conserved motif of MYB was determined by MEME (https://meme-suite.org/meme/, accessed on 12 October 2022) [60], with an optimal motif length of 6–200 residues and a search number of 10. The conservative DBD characteristics of MYB were analyzed using WebLogo (http://weblogo.berkeley.edu/logo.cgi, accessed on 1 October 2022) [61]. All software was used under default parameter settings.

### 4.4. MYB Sequence Alignment and Phylogenetic Analysis

Muscle was used for multiple sequence alignment of MYBs. MEGAX v7.0.14 was used to explore the evolutionary relationship between the MYB sequences of *A. thaliana* as a reference sequence [62]. A phylogenetic tree (ML) was constructed using Muscle to compare the full lengths of MYB amino acid sequences (184 *R. delavayi* MYBs and 132 *A. thaliana* MYBs). Parameters were set to WAG + G, paired deletion, and bootstrap analysis with 1000 bootstrap replicates. *R. delavayi* MYBs were classified based on the phylogenetic relationship with the corresponding classification of MYBs in *A. thaliana*.

### 4.5. Analysis of Promoter Cis-Acting Elements of MYB

The upstream 2000 bp sequence of *MYB* CDSs of *R. delavayi* was extracted from the RPGD as promoters [28]. Putative binding sites for TF candidates were determined using the PlantPAN3.0 server (http://plantpan.itps.ncku.edu.tw/, accessed on 9 February 2023) and *A. thaliana* as references [63].Cis elements of the promoters were predicted using PlantCARE [64] to identify the three main types of cis-acting elements on the promoter region: phytohormone response, including Me-JA response element (CGTCA and TGACG motifs), ABRE, gibberellin response element (P-box), and salicylic acid response element (TCA element); biotic/abiotic stress response, including ARE, LTR, and drought induction element (MBS); plant growth and development, including light response element (G-Box), growth hormone response element (TGA element), defense and stress response element (TC-rich repeats), trauma response element (WUN-motif), and flavonoid biosynthesis (MBSI). Statistical analysis and cis-element visualization were performed using Excel and TBtools, respectively [59].

### 4.6. Transcriptome Analysis of Five Types of Red Samples

Total RNA was extracted from samples using the RNAprep Pure Polysaccharide Polyphenol Plant Kit (TianGen, Beijing, China). The qualified RNA samples were sent to Novogene Bioinformatics Technology Co., Ltd. (Beijing, China). The libraries were sequenced on Illumina Novaseq 6000 Platform and generated 150 nt pair-end reads. Raw reads were first processed through in-house perl scripts. Clean reads were obtained by removing reads containing adapter and/or ploy-N, and low-quality reads from raw data. At the same time, Q20, Q30, and GC content of the clean data were calculated. UMI sequences on each read were identified with UMI-tools (1.0.0) [65]. Genome of *R. delavayi* (http://bioinfor.kib.ac.cn/RPGD/index.html, accessed on 1 October 2022) [28,66] was used as reference to assemble the transcriptome using HISAT2 v2.0.4 [67]. The number of reads mapped to each gene was calculated using HTSeq v0.6.1 [68]. The FPKM of each gene was calculated based on the gene length and counts mapped to the corresponding gene.

### 4.7. Analysis of DEGs, GO, and KEGG 

DESeq R package (1.10.1) was used to analyze the differential expression between two conditions/groups (two biological replicates per condition). Corrected *p*-value of 0.005 and log_2_ (fold change) of 1 were set as the threshold for significance. GO analysis of DEGs was implemented by the GOseq R package, in which gene length bias was corrected. GO terms with corrected *p*-value less than 0.05 were considered significantly enriched by DEGs. In KEGG enrichment analysis, KOBAS 3.0 (http://kobas.cbi.pku.edu.cn/index.php, accessed on 20 May 2022 ) database was used to test the statistical enrichment of DEGs.

### 4.8. Expression Analyses of R2R3-MYB Genes in R. delavayi

The transcript abundances of *R. delavayi* R2R3-MYB genes were calculated with FPKM values. Heatmaps of R2R3-MYB gene expression data were generated using TBtools v1.0987663 [59]. Gene expression levels were expressed by log_2_ FPKM values.

### 4.9. Analysis of Weight Co-Expression Network

WGCNA was performed using the R v1.63 software package. Genes with coefficient of variation less than 0.5 were removed, and cluster trees were constructed based on the correlation and gene expression levels. In the WGCNA network, the soft threshold was set to 5 (Figure S2), the module size was set to at least 30, and the merge cut height value was set to 0.25 (Figure S3). The correlation between the modules and the chromatic aberration values of the five types of red samples was analyzed for all genes in each module. The significant trait correlation module was identified based on the high correlation values and significance (*p*-value). Finally, Cytoscape v3.9.1 [69] was used to display the weight co-expression network of the selected modules, calculate the degree values of the whole network, and screen the hub genes with high connectivity.

### 4.10. qRT-PCR Verification

Expression patterns of nine selected genes were verified by qRT-PCR. The qualified RNA (1 µg) was reverse transcribed using TRUEscript RT Kit with gDNA clearance (Aidlab, Beijing, China). Specific primers were designed by Primer v6.0 (ref), and 18S was used as an internal control [70] (Table S6). Three biological replicates and three technological replicates of qRT-PCR were performed for verification. Each reaction contained 10 µL SYBR qPCR Mix (Aidlab, China), 1 µL cDNA, 10.5 µL distilled water, 0.5 µL forward primers, and 0.5 µL reverse primers under the following conditions: 94 °C for 2 min, 40 cycles; 94 °C for 30 s; 60 °C for 20 s; 72 °C for 30 s; 4 °C. The relative expression level of genes was calculated using 2^−ΔΔct^.

## 5. Conclusions

In this study, 184 *MYB* genes were identified in the *R. delavayi* genome and classified into 35 subgroups based on phylogenetic analyses. Bioinformatics analysis was performed including, conserved domains and motifs, gene structures, promoter cis-acting elements, and transcriptomic profiling. In addition, the expression levels of the R2R3-MYB gene were significantly different in five types of red samples. WGCNA between transcriptome and chromatic aberration values showed that 10 *MYBs*, six *bHLHs*, one *WD40*, two MYB-related genes, and four structural genes *UDPGTs* constructed the co-expression network behind the coloration in *R. delavayi*. The MYBs were the most important transcription factors involved in the red color formation, among them, two R2R3-MYB (DUH019226.1 and DUH019400.1) were identified as hub genes. Furthermore, the qRT-PCR validated the results of the transcriptome. This work has enhanced a comprehensive understating of the MYB gene family in *R. delavayi* and provided references for the study of transcriptional regulation of the red color formation of *R. delavayi*.

## Figures and Tables

**Table 1:** Chromatic aberration values of different tissues of *R. delavayi*.

Samples	L*	a*	b*
MY-1	32.26 ± 1.37 b	57.90 ± 3.98 b	4.37 ± 1.39 b
MY-2	36.25 ± 0.29 a	64.23 ± 2.27 a	7.86 ± 0.61 a
MY-3	29.28 ± 1.53 c	39.83 ± 4.39 c	1.99 ± 1.12 c
MY-4	37.79 ± 1.13 a	53.02 ± 1.10 b	3.18 ± 0.76 bc
MY-5	32.08 ± 1.14 b	10.23 ± 2.74 d	8.58 ± 1.30 a

Significant differences (*p* < 0.05) are indicated by different letters.

## Data Availability

*R. delavayi* genome data can be searched in open access RPGD database (http://bioinfor.kib.ac.cn/RPGD/index.html, accessed on 1 October 2022). The raw data of transcriptome has been uploaded to the Sequence Reading Archive database (http://www.ncbi.nlm.nih.gov/sra/, accessed on 3 December 2022) with the entry number PRJNA907866. Additional data presented in this study are presented in the supplementary materials.

## Supplementary Materials

The following supporting information can be downloaded at: https://www.mdpi.com/1422-0067/24/5/4641/s1.
