# Supplementary material for: Genome-Wide Analysis of MYB Transcription Factors and Screening of MYBs Involved in the Red Color Formation in Rhododendron delavayi
Source: Int J Mol Sci. 2023 Feb 28;24(5):4641. doi: 10.3390/ijms24054641 (PMC10037418; doi:10.3390/ijms24054641)
Supplement: Supplementary file 1 [file ijms-24-04641-s001.zip › Figure S2.pdf]

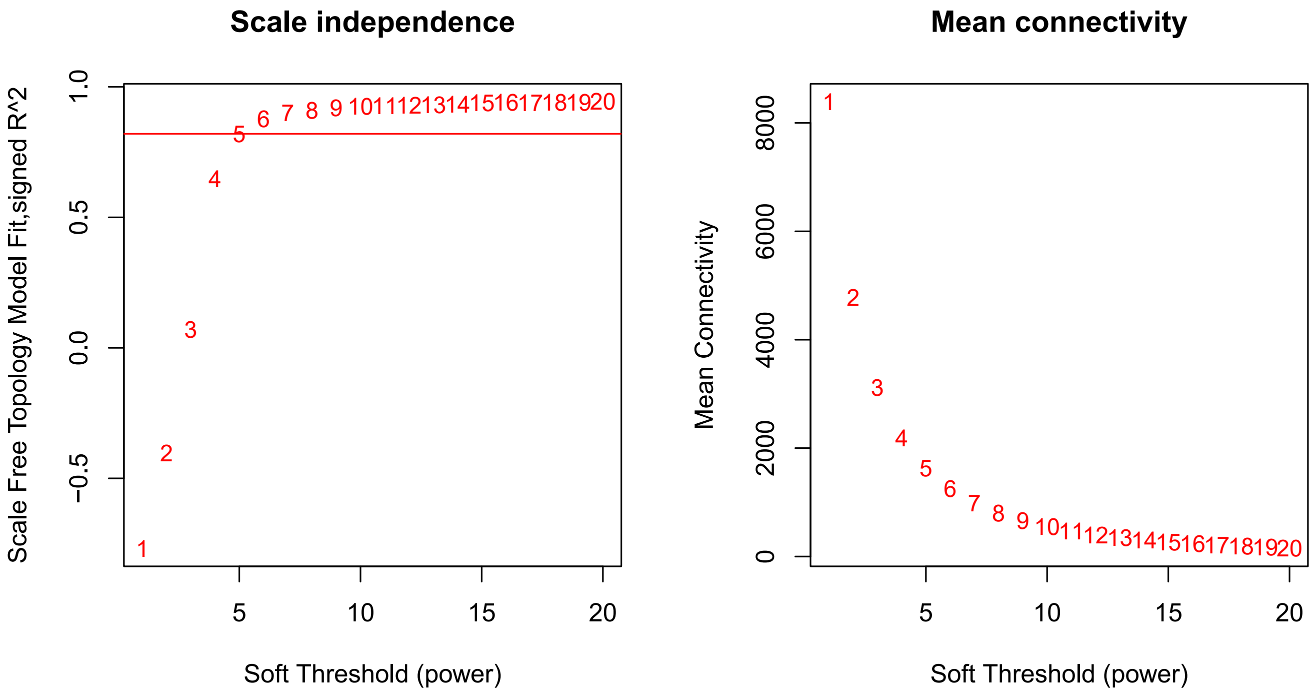

Figure S2 Soft threshold (β value) determination that makes the network close to scale-free; the red dotted line is drawn at 5.
