# Supplementary material for: Genome-Wide Analysis of MYB Transcription Factors and Screening of MYBs Involved in the Red Color Formation in Rhododendron delavayi
Source: Int J Mol Sci. 2023 Feb 28;24(5):4641. doi: 10.3390/ijms24054641 (PMC10037418; doi:10.3390/ijms24054641)
Supplement: Supplementary file 1 [file ijms-24-04641-s001.zip › Figure S3.pdf]

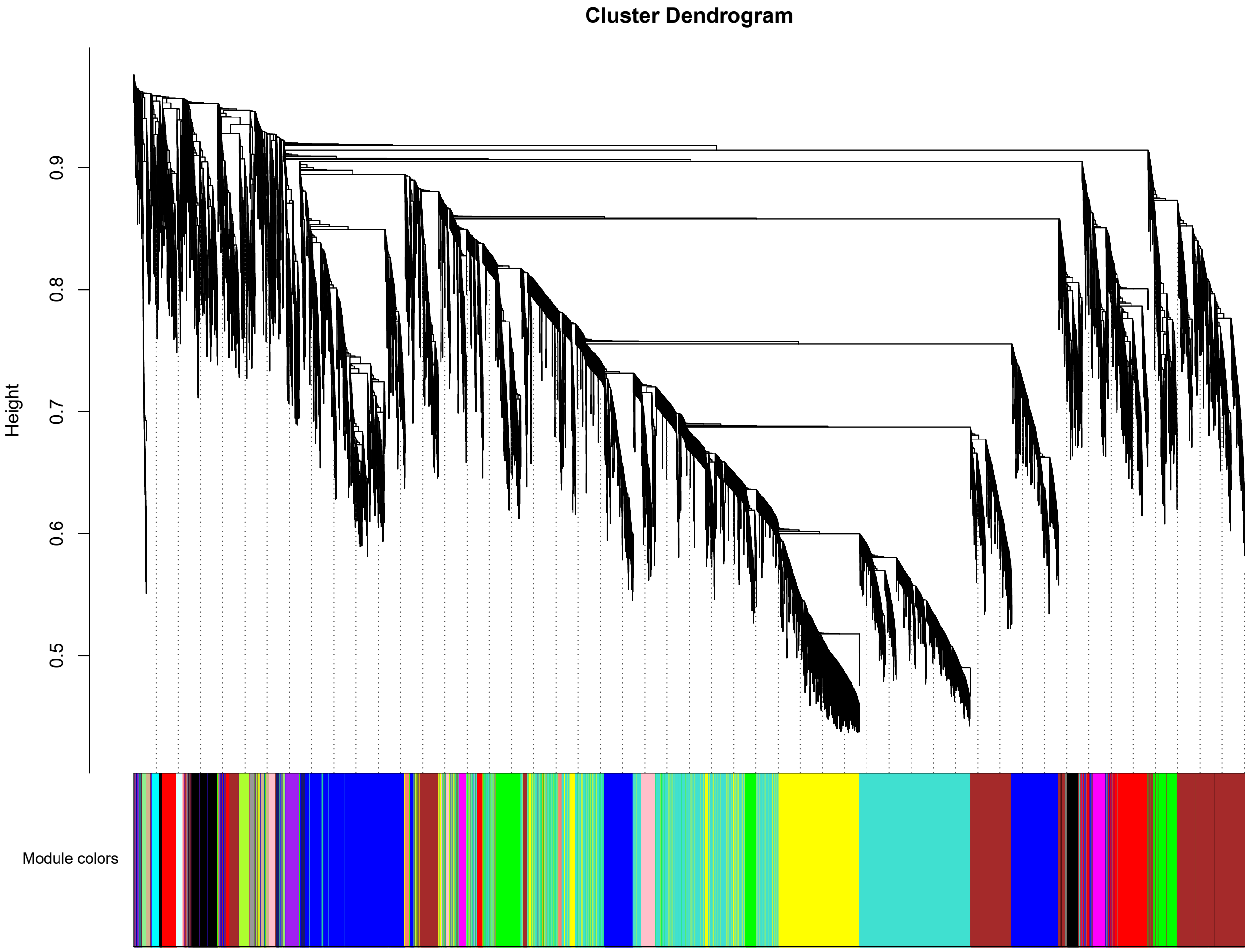

Figure S3 The genes were clustered, and then the tree was clipped into different modules using the dynamic shearing method (the minimum number of genes in themodule was set to 30, and the merge cut height value was set to 0.25)
