# Supplementary figures and images for: Genome-Wide Analysis of MYB Transcription Factors and Screening of MYBs Involved in the Red Color Formation in Rhododendron delavayi
Source: Int J Mol Sci. 2023 Feb 28;24(5):4641. doi: 10.3390/ijms24054641 (PMC10037418; doi:10.3390/ijms24054641)

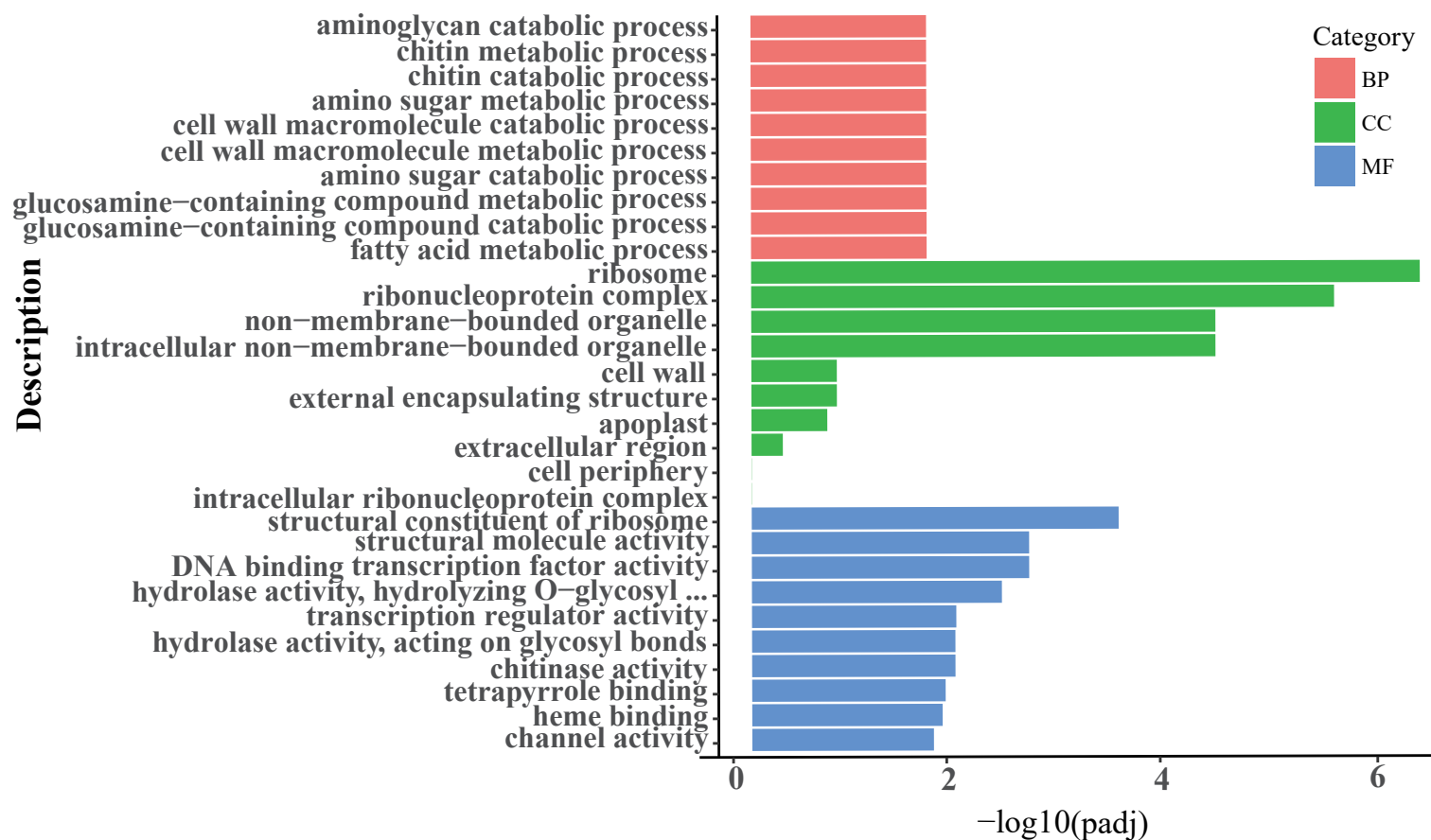

Figure S1. GO enrichment analysis of MY-1 vs MY-2 DEGs. MY-1, spotted petals; MY-2, unspotted petals;

Supplement: Supplementary file 1 [file ijms-24-04641-s001.zip › Figure S1.pdf]
